# Cultural Adaptation and Measurement Properties of the Iranian Version of the Families' Importance in Nursing Care—Nurses Attitudes Questionnaire Based on COSMIN Checklist: A Methodological Study

**DOI:** 10.1002/mpr.70093

**Published:** 2026-06-30

**Authors:** Amirmohammad Dahouri, Mina Hosseinzadeh, Mohammad Hassan Sahebihagh, Fereshteh Mostafazadeh Meinag

**Affiliations:** ^1^ Department of Community Health Nursing Faculty of Nursing and Midwifery Research Center of Psychiatric and Behavioral Sciences Razi Hospital Tabriz University of Medical Sciences Tabriz Iran; ^2^ Department of Community Health Nursing Faculty of Nursing and Midwifery Tabriz University of Medical Sciences Tabriz Iran; ^3^ Department of Community Health Nursing, Tabriz Health Services Management Research Center Tabriz University of Medical Sciences Tabriz Iran; ^4^ Tabriz Medical Sciences Islamic Azad University Tabriz Iran

**Keywords:** COSMIN checklist, family‐centered care, iranian validation, nursing attitudes, psychometric, reliability

## Abstract

**Background:**

Family involvement in nursing care is a key component of patient‐centered care and is associated with improved patient outcomes and satisfaction. However, in Iran, there is a need for a culturally adapted and psychometrically sound instrument to assess nurses' attitudes toward family involvement in care using a standardized framework.

**Objective:**

To adapt and evaluate the psychometric properties of the Persian version of the Families' Importance in Nursing Care—Nurses’ Attitudes (FINC‐NA) questionnaire among Iranian nurses based on the COSMIN framework.

**Method:**

This cross‐sectional methodological study was conducted among 430 nurses from Tabriz educational hospitals between January and March 2025. Participants were randomly divided into exploratory (*n* = 215) and confirmatory (*n* = 215) factor analysis groups. The Persian FINC‐NA was evaluated for content and face validity, construct validity (EFA and CFA), and reliability (internal consistency and test–retest reliability) following COSMIN guidelines. Data were analyzed using SPSS version 27 and AMOS version 24.

**Results:**

Exploratory and confirmatory factor analyses supported a four‐factor structure of the Persian FINC‐NA: Family as an Active Care Partner, Family Engagement and Support, Family Burden, and Family Strengths and Communication. This structure explained 56.65% of the total variance and demonstrated acceptable model fit indices (*χ* ^2^/df = 3.63, CFI = 0.901, TLI = 0.949, RMSEA = 0.078, SRMR = 0.069). Internal consistency was acceptable for most subscales (*α* ≥ 0.83), and test–retest reliability ranged from poor to good across subscales (ICC = 0.158–0.839).

**Conclusion:**

The Persian version of the FINC‐NA demonstrates acceptable validity and reliability for assessing nurses' attitudes toward family involvement in nursing care in Iran. However, further refinement of certain subscales may be required to improve stability over time.

## 1 Introduction

In recent decades, the involvement of patients' families in healthcare delivery has gained increasing attention across clinical and research settings (Adams et al. 2017; Dees et al. 2022; Duque‐Ortiz and Arias‐Valencia 2020). Nurses' interactions with families are now recognized as an important component of high‐quality care, particularly in contexts where family members actively participate in supporting patients during illness and recovery. Research on this topic has been conducted across a wide range of clinical environments, including intensive care units, surgical wards, internal medicine departments, pediatric settings, palliative care services, and long‐term care facilities, reflecting its broad relevance in nursing practice (Aass et al. 2022; Chen et al. 2021; Mabunda 2024; Mæhre et al. 2023; Mcharo et al. 2023).

Evidence suggests that although nurses generally acknowledge the importance of families in patient care, this recognition is not always consistently translated into clinical practice (Alfaro‐Diaz et al. 2024; Verkaik et al. 2025). Nurses' attitudes toward family involvement are often complex and multidimensional, shaped by both facilitating and limiting beliefs (Barreto et al. 2022; Cranley et al. 2022). These attitudes may influence the extent to which families are included in care processes, communication, and decision‐making, thereby affecting the overall delivery of family‐centered care.

Given the importance of this topic, several instruments have been developed to assess nurses' attitudes and perceptions toward family involvement in different clinical contexts (Benzein et al. 2008; Sawin 2016). These tools have been applied to examine nurses' views in areas such as critical care, emergency situations, pediatric care, and end‐of‐life settings, as well as to identify perceived barriers and facilitators of family participation (Alfaro‐Diaz et al. 2024; Cranley et al. 2022; Sawin 2016). Collectively, these measurement approaches highlight the need for reliable assessment of nurses' attitudes as a foundation for improving family‐centered nursing practice.

Family involvement is widely considered a key element of holistic nursing care (Maragakis et al. 2021). Families contribute not only to emotional and physical support for patients but also to communication, care continuity, and shared decision‐making throughout the course of illness (Beach et al. 2021). Previous studies have shown that more positive nursing attitudes toward family involvement are associated with improved patient outcomes, higher satisfaction levels, and stronger collaboration between healthcare providers and families (Alfaro‐Diaz et al. 2024; Cranley et al. 2022). However, these attitudes are not uniform and may vary depending on cultural expectations, organizational structures, and professional education (Shamali et al. 2023).

Among existing instruments, the Families' Importance in Nursing Care—Nurses’ Attitudes (FINC‐NA) questionnaire is one of the most widely used measures for assessing nurses' perspectives on family involvement (Benzein et al. 2008). Developed in Sweden, the FINC‐NA captures multiple dimensions of nurses' attitudes, including perceptions of family support, collaboration, burden, and importance in care (Benzein et al. 2008). This instrument has been translated and validated in several countries and has demonstrated acceptable psychometric properties across different cultural settings (Hagedoorn et al. 2018; Naef et al. 2021; Pascual Fernández et al. 2015).

Despite its wide application, direct use of instruments developed in other cultural contexts may not adequately reflect local values, healthcare structures, and clinical practices (Martin and Savage‐McGlynn 2013; Weck and Ivanova 2013). Cultural differences can significantly influence how family involvement is perceived and practiced in nursing care (Epstein et al. 2015). Therefore, cross‐cultural adaptation is essential to ensure both linguistic accuracy and conceptual equivalence of measurement tools in new settings (Mokkink et al. 2012). In addition, rigorous psychometric evaluation is required to confirm that adapted instruments are valid, reliable, and suitable for use in both clinical and research contexts.

In Iran, family members play a central role in patient care due to strong cultural and social traditions that emphasize family responsibility and support (Tehranineshat et al. 2020). However, despite this prominent role, there is a lack of validated Persian‐language instruments specifically designed to assess nurses' attitudes toward family involvement in nursing care. This gap limits the ability to systematically evaluate attitudes and to develop evidence‐based strategies for strengthening family‐centered care within Iranian healthcare settings.

Therefore, the present study aimed to culturally adapt the FINC‐NA questionnaire for use in Iran and to evaluate its psychometric properties in accordance with COSMIN (COnsensus‐based Standards for the selection of health Measurement INstruments) guidelines. This study seeks to provide a culturally appropriate, reliable, and valid instrument for assessing nurses' attitudes toward family involvement in care, thereby contributing to the advancement of family‐centered nursing practice in Iran.

## 2 Method

### 2.1 Ethical Consideration and Consent to Participate

Ethical approval for this study was obtained from the Ethics Committee of Tabriz University of Medical Sciences (Approval No: IR.TBZMED.REC.1403.332). Additional administrative permissions were granted by the Research Council and the Vice‐Chancellor for Research at the Faculty of Nursing and Midwifery. The study was conducted in accordance with the ethical principles of the Declaration of Helsinki.

Written informed consent was obtained from all participants prior to data collection. Participation was voluntary, and nurses were informed about the study objectives and procedures, as well as their right to withdraw at any time without any consequences. Eligible participants were approached by the researchers, and questionnaires were completed in a supervised setting to ensure clarity and completeness.

### 2.2 Study Design and Validation Process

This cross‐sectional methodological study was conducted to evaluate the measurement properties of the Iranian version of the Families' Importance in Nursing Care—Nurses' Attitudes (FINC‐NA) questionnaire, following the guidelines of the COSMIN checklist for health measurement instruments (Mokkink et al. 2012). This study adhered to the EQUATOR Network recommendations. The evaluation process included translation and cultural adaptation, assessment of content validity, face validity, construct validity (through exploratory and confirmatory factor analyses, convergent, and divergent validity), reliability (internal consistency and test–retest reliability), measurement error, and examination of responsiveness, interpretability, and floor and ceiling effects.

The study was carried out between 20 January and 19 March 2025 among nurses working in government hospitals affiliated with Tabriz University of Medical Sciences, located in Tabriz, Iran. A total of 430 nurses were initially approached. We have no attrition rate so response rate of our study was 100% (430/430). For psychometric assessment, the sample was divided into two subgroups: 215 participants for exploratory factor analysis (EFA) and 215 participants for confirmatory factor analysis (CFA). Our Inclusion and Exclusion Criteria were: Eligible participants were nurses who had at least 6 months of work experience, were employed full‐time in clinical departments (e.g., internal medicine, emergency, intensive care), and provided informed consent. Nurses were excluded if they: were not actively working in clinical settings, held only administrative or non‐clinical roles, or declined to provide informed consent.

A stratified random sampling method was employed in this study, with each hospital considered a separate stratum. The participating hospitals included Sina (*n* = 372), Alzahra (*n* = 179), Emam Reza (*n* = 858), Razi (*n* = 243), Shohada (*n* = 186), Shahid Madani (*n* = 437), Taleghani (*n* = 103), Alavi (*n* = 27), Nikoukari (*n* = 42), and Mardani Azar (*n* = 480). These figures, representing the total number of nurses employed at each hospital, were obtained from the Office of the Vice Chancellor for Health at Tabriz University of Medical Sciences.

Using a proportional allocation approach, the number of nurses selected from each hospital was determined based on the relative size of the nursing workforce at each site, in accordance with the final calculated total sample size of 430 (as described in the sample size determination section). Accordingly, the sample distribution was as follows: Emam Reza (*n* = 127), Mardani Azar (*n* = 71), Shahid Madani (*n* = 65), Shohada (*n* = 27), Razi (*n* = 35), Alzahra (*n* = 26), Sina (*n* = 55), Taleghani (*n* = 15), Nikoukari (*n* = 6), and Alavi (*n* = 3).

The total number of nurses in each hospital was obtained from official administrative records provided by the Nursing Office of Tabriz University of Medical Sciences (under the Vice Chancellor for Medical Affairs). These records reflected the most recent workforce statistics available at the time of sampling.

From these lists, individual participants were selected randomly using the online tool www.random.org After selection, the nurses were contacted by telephone. During the call, the researchers provided a brief overview of the study and invited them to attend an in‐person meeting at their workplace within a specified timeframe.

At the meeting, participants were first screened based on the inclusion and exclusion criteria. Those who met the eligibility requirements received comprehensive information about the study's objectives, procedures, and ethical considerations, including assurance of confidentiality. Nurses who agreed to participate provided oral informed consent, in accordance with the approval of the university's ethics committee and the principles of the Declaration of Helsinki. They then completed the socio‐demographic questionnaire and the FINC‐NA instrument.

It is important to note that random selection was performed prior to eligibility screening, and participation was arranged during nurses' available working hours to ensure that their clinical responsibilities were not disrupted.

Data collection was conducted by a trained research team composed of two investigators. The first author (AMD) and FMM were directly involved in participant recruitment, eligibility screening, explanation of study objectives, and administration of questionnaires. Data collection was performed in supervised face‐to‐face sessions within the clinical setting to ensure completeness and minimize response errors. All questionnaires were checked immediately upon completion, and participants were asked to clarify any missing or ambiguous responses at the time of completion. In addition, completed forms were reviewed again by the data collection team before data entry to ensure accuracy and completeness. Data curation and final dataset preparation were performed by FMM and AMD prior to statistical analysis.

### 2.3 Translation Process

Prior to initiating the translation process, formal permission to use the original FINC‐NA instrument was obtained from the original instrument developer. After confirming that no validated Persian version existed, permission for translation and cultural adaptation was granted via email (Benzein et al. 2008).

The translation and cross‐cultural adaptation of the FINC‐NA into Persian were conducted in accordance with internationally recognized guidelines, including those proposed by the World Health Organization (WHO), the European Organisation for Research and Treatment of Cancer (EORTC) Quality of Life Group (Koller et al. 2006; WHO 2015), and the 10‐step procedure recommended by (Wild et al. 2005). The process integrated both forward–backward translation and a dual‐panel approach and was completed in four stages: forward translation, backward translation, cognitive debriefing, and finalization.

In the first stage, the original English version of the FINC‐NA was independently translated into Persian by two bilingual translators whose native language was Persian and who were fluent in English and familiar with nursing terminology and instrument development. Translators were instructed to prioritize conceptual equivalence over literal translation and to ensure clarity and comprehensibility for the target population. Discrepancies between the two forward translations were resolved through discussion, resulting in a single reconciled Persian version.

The reconciled version was then back‐translated into English by two independent translators whose native language was English and who were blinded to the original instrument and the forward translation. The back‐translated versions were compared with the original instrument by the research team to identify and resolve any discrepancies or conceptual inconsistencies, ensuring semantic and conceptual equivalence.

The full translation package, including forward translations, reconciled version, back‐translations, and accompanying documentation, was reviewed by an expert panel consisting of specialists in nursing, psychometrics, and instrument development. This panel evaluated semantic, conceptual, and cultural equivalence and provided recommendations for refinement to ensure contextual appropriateness for the Iranian nursing setting.

To assess face validity and cultural relevance, a pilot study was conducted with a purposive sample of nurses from the target population. Participants completed the preliminary Persian version of the FINC‐NA and provided structured feedback regarding clarity, wording, grammar, and comprehensibility. Based on their feedback, minor revisions were made to improve clarity and usability. The final version was then prepared for psychometric evaluation.

Throughout the process, the 10‐step guideline for translation and cultural adaptation proposed by Wild et al. (2005) (Wild et al. 2005) was systematically followed, including preparation, forward translation, reconciliation, back‐translation, expert review, cognitive debriefing, analysis of feedback, proofreading, and final documentation of the translation procedure. This rigorous process ensured that the Persian version of the FINC‐NA maintained conceptual equivalence, cultural relevance, and psychometric integrity for use in Iranian nursing populations (Kalfoss 2019).

### 2.4 Measures

In this study, two questionnaires were used as follows:

#### 2.4.1 Demographic and Work Environment Variables

The first section of the data collection instrument included demographic characteristics and work environment conditions. Demographic variables collected were age, gender, and level of education. Work environment variables included hospital ward (e.g., internal medicine, emergency, intensive care), weekly working hours, years of nursing experience, awareness of organizational policies regarding family involvement in care, and previous training in family nursing. In the context of this study, all demographic and work‐related variables were treated as potential confounding variables.

#### 2.4.2 Nurses' Attitudes Toward the Importance of Families in Nursing Care (FINC‐NA)

To assess nurses' attitudes toward family involvement in nursing care, the Families' Importance in Nursing Care—Nurses' Attitudes (FINC‐NA) questionnaire, developed by (Benzein et al. 2008), was utilized. The FINC‐NA comprises 26 items across four theoretically derived subscales: 1‐Family as a Resource in Nursing Care (Fam‐RNC)—10 items assessing the perceived importance of involving families in patient care activities. 2‐Family as a Conversational Partner (Fam‐CP)—8 items reflecting the value of engaging with family members as communicative partners in care. 3‐Family as a Burden (Fam‐B)—4 items expressing the perception of families as hindering nursing work. 4‐Family as a Resource to the Own Wellbeing (Fam‐OR)—4 items addressing the family's potential role in supporting nurses' own professional sense of meaning and satisfaction.

Responses are recorded on a five‐point Likert scale, ranging from 1 (Strongly disagree) to 5 (Strongly agree). Total scores range from 26 to 130, with higher scores indicating more supportive attitudes toward family involvement in nursing care. Items within the Fam‐B subscale are negatively worded and were reverse scored prior to analysis to ensure consistency in interpretation across the scale.

The FINC‐NA has demonstrated satisfactory psychometric properties in its original Swedish version and in several translated versions. In this study, the instrument underwent rigorous translation and validation procedures to ensure cultural relevance and psychometric adequacy for the Persian‐speaking nursing population.

It should be noted that the FINC‐NA questionnaire (originally developed in Swedish) has been translated and psychometrically tested in several countries. For example, Spanish, Portuguese (European and Brazilian), Dutch, and German versions have been reported, each demonstrating acceptable reliability and supporting (or modifying) the original factor structure.

Spain (Spanish version): Conducted a translation and validation of the FINC‐NA for Spanish nurses (*n* = 274). They found good internal consistency (total scale Cronbach's *α* = 0.864; subscales 0.769–0.888) and a four‐factor structure similar to the original, explaining 54% of the variance (Pascual Fernández et al. 2015).

Portugal (European Portuguese): Adapted FINC‐NA for Portuguese nurses (*n* = 136) and reported satisfactory reliability (Cronbach's *α* = 0.87). They concluded the Portuguese FINC‐NA (called IFCE‐AE) is a valid, reliable tool for assessing nurses' family‐involvement attitudes (Oliveira Pda et al. 2011).

Brazil (Brazilian Portuguese): In a study (*n* = 283) adapted FINC‐NA to Brazilian culture. After exploratory/confirmatory factor analysis, five items were dropped and three factors (reinterpreted theoretically) were retained (18 items total), with strong internal consistency (Cronbach's *α* = 0.91). The authors noted satisfactory construct validity and reliability for this 18‐item, three‐factor Brazilian version (Ruiz et al. 2022).

Netherlands (Dutch version): Tested the Dutch FINC‐NA in 597 nurses using Rasch/partial credit modeling. They confirmed the original four‐subscale structure and found that most items worked well (monotonicity, discrimination). (Cronbach's *α* was not reported due to the modeling approach, but the four‐factor structure was upheld) (Hagedoorn et al. 2018).

Germany/Switzerland (German version): Translated and tested FINC‐NA with 316 acute/critical care nurses. They largely confirmed the four‐factor model (families as partner, resource, own resource, burden) but removed seven items for weak loadings. The final 19‐item German FINC‐NA had subscale alphas ranging from 0.68 to 0.86. Overall, these adaptations show that the FINC‐NA retains good internal consistency and broadly similar factor structures in different languages, with minor modifications (item removals or factor reconfiguration) in some contexts (Naef et al. 2021).

### 2.5 Sample Size Determination

An appropriate sample size is essential to ensure the reliability and validity of factor analysis outcomes. According to established guidelines, sample size adequacy for EFA is classified as follows: 50 participants is considered very poor, 100 poor, 200 fair, 300 good, 500 very good, and 1000 excellent (Comrey and Lee 2013). According to Gorsuch et al. (1997), a sample size of 5 participants per item is recommended to enhance the generalizability and statistical precision of a study's findings. Conversely, (J. Nunnally and Bernstein 1994) recommends recruiting a larger sample of 10 participants per item on a measurement instrument. Given that the FINC‐NA questionnaire comprises 26 items, a ratio of 10 participants per item was selected, establishing a minimum required sample size of 260. Since this study employed stratified proportional random sampling, an adjustment was made for the design effect (Deff)—a critical factor in sampling designs that involve stratification. In stratified sampling, the design effect is typically less than 1, indicating increased statistical efficiency due to reduced variance within strata (Kish 1995). However, to account for potential stratifying effects and to ensure conservative estimation, a design effect of 1.5 was applied, in line with common recommendations where Deff values typically range between 1.5 and 2 (Alimohamadi and Sepandi 2019; J. C. Nunnally and Bernstein 1994). Furthermore, an additional 10% was added to the calculated sample to account for potential non‐responses or dropouts. As a result, the final estimated sample size required for this study was 430 participants.

### 2.6 Methodological Testing Based on COSMIN Checklist

#### 2.6.1 Validity

Validity refers to the degree to which an instrument precisely captures the construct it is intended to measure (Martin‐Key et al. 2022).

##### 2.6.1.1 Face Validity Assessment

Face validity refers to the extent to which the items of an instrument appear to be relevant, clear, and representative of the construct being measured, as perceived by members of the target population (Juniper et al. 1999). It assumes that the intended respondents share the researcher's understanding of the instrument's purpose and wording. For a tool to demonstrate strong face validity, its items should be clearly worded, logically sequenced, visually accessible (e.g., font and formatting), and easy to interpret, so that respondents do not experience confusion or hesitation while completing it (Mokkink et al. 2012).

In this study, face validity was evaluated using both qualitative and quantitative approaches. For the qualitative assessment, a sample of 20 nurses from hospitals in Tabriz City was selected through convenience sampling. These nurses reviewed the preliminary version of the Persian FINC‐NA questionnaire and provided feedback on item clarity, difficulty, ambiguity, and relevance.

For the quantitative assessment, item impact scores were calculated to determine the perceived importance of each item. Participants rated each item on a 5‐point Likert scale, ranging from 1 (not important at all) to 5 (completely important). The impact score was computed using the formula:

ImpactScore=Frequency(%)×Importance

In this formula, frequency refers to the percentage of nurses who rated the item as either “important” (score 4) or “completely important” (score 5). Importance denotes the mean score of all responses for that item, based on the 5‐point Likert scale (ranging from 1 to 5). An impact score greater than 1.5 reflects an acceptable level of perceived importance among participants, assuming that at least 50% of respondents rated the item as important and the average importance rating was no less than 3. This threshold is widely used in face validity assessments to determine which items are sufficiently valued by the target population to be retained for further psychometric testing (Seyf 2016).

##### 2.6.1.2 Content Validity Assessment

Content validity refers to the extent to which the items of a health‐related patient‐reported outcome (HR‐PRO) instrument adequately reflect the construct it is intended to measure (Mokkink et al. 2012). In this study, both qualitative and quantitative approaches were employed to evaluate the content validity of the Persian version of the FINC‐NA questionnaire. Specifically, content validity was assessed using two established metrics: the Content Validity Ratio (CVR) and the Content Validity Index (CVI) (Yang et al. 2023).

The evaluation of CVR was conducted in two phases: a qualitative review and a quantitative scoring. In the qualitative phase, a panel of 12 subject matter experts (SMEs) was recruited. The panel comprised one expert in midwifery, two in pediatric nursing, five in medical‐surgical nursing, three in community health nursing, and one in mental health nursing. All experts held a PhD in nursing or midwifery, were academic faculty members with at least the rank of assistant professor, and had clinical experience across various hospital settings. These experts assessed each item for clarity, Persian grammar, vocabulary appropriateness, item sequencing, and the comprehensibility of response options. Their suggestions were incorporated to enhance the linguistic and cultural accuracy of the translated instrument.

In the quantitative phase, the same experts were asked to rate the necessity of each item using a 4‐point Likert scale: 1 = necessary, 2 = needs revision, 3 = useful but not necessary, 4 = not necessary. For analysis, responses marked as “necessary” or “needs revision” were recoded as necessary (code = 1), while “useful but not necessary” and “not necessary” were recoded as not necessary (code = 0). The CVR for each item was calculated using the following formula:

CVR=(nE−N/2)/(N/2)

Where nE is the number of experts who rated the item as necessary, and *N* is the total number of experts (in this study, 12) (Lawshe 1975).

To determine the statistical significance of CVR values, Lawshe's critical value table was used as the primary reference, particularly as updated and validated by Ayre and Scally (2014), who confirmed its accuracy and reliability (Ayre and Scally 2014). For a panel of 12 experts, the minimum acceptable CVR value was set at 0.54 according to Lawshe's original criteria, and 0.53 based on the interpretation by Zareiyan (Geranmayeh et al. 2020). Both thresholds correspond to a one‐tailed test with a significance level of *p* < 0.05. Given the existing criticisms of Lawshe's method in the literature (Tristán‐López 2008; Wilson et al. 2012), we employed both approaches—Lawshe's original and Zareiyan's interpretation—to interpret CVR results.

The CVI, originally developed by Waltz and Bausell (Waltz and Bausell 1981), was assessed using both qualitative and quantitative methods. The evaluation involved a two‐phase process focusing on three key dimensions: relevance, clarity, and simplicity.

In the qualitative phase, a panel of 12 SMEs—previously introduced in the CVR section—reviewed each item in the Persian version of the questionnaire. They provided feedback on the appropriateness of item wording, clarity of expression, simplicity, and conceptual relevance. Their recommendations were incorporated into the revised version of the questionnaire.

Following this, the quantitative phase was conducted. Experts rated each item across the three dimensions (relevance, clarity, and simplicity) using a 4‐point Likert scale:

1 = not relevant/not simple/not clear, 2 = needs major revision, 3 = relevant/simple/clear but needs minor revision, 4 = completely relevant/simple/clear. For analysis purposes, scores of 3 and 4 were recoded as “acceptable” (coded as 1), while scores of 1 and 2 were recoded as “not acceptable” (coded as 0) for each item per dimension. The Item‐level CVI (I‐CVI) was then calculated as the proportion of experts rating the item as acceptable (scores 3 or 4) using the following formula:

$$CVI=\frac{number\;of\;raters\;giving\;a\;rating\;of\;3\;or\;4}{total\;number\;of\;raters}$$

According to Polit et al. (2007), the I‐CVI for each item was interpreted as follows: I‐CVI ≥ 0.79: Item is acceptable, I‐CVI between 0.70 and 0.79: Item requires revision, and I‐CVI < 0.70: Item should be eliminated.

In addition to item‐level indices, two scale‐level CVIs were calculated: S‐CVI/Ave (average congruency percentage): the average of all I‐CVI values across items.

S−CVI/Ave=(∑░〖I−CVI〗)/(numberofitems)

A value of ≥ 0.90 is generally considered acceptable for new instruments (Polit et al. 2007).

S‐CVI/UA (universal agreement): the proportion of items that achieved universal agreement (i.e., all 12 experts rated the item as acceptable).

S−CVI/UA=(numberofitemswithI−CVI=1)÷(totalnumberofitems)

This measure is more conservative and is often used alongside S‐CVI/Ave.

To address the limitation of chance agreement among raters, the modified kappa statistic K* was calculated for each item, as recommended by Polit et al. (2007) and Wynd et al. (2003). First, the probability of chance agreement (Pc) was computed using the formula:

Pc=[N!/A!(N−A)!]×〖0.5〗^N

Where *N* is the number of experts and A is the number of experts who agreed the item was acceptable (i.e., rated it 3 or 4).

Then, the modified kappa (K*) was calculated as:

K^∗=(I−CVI−Pc)/(1−Pc)

Interpretation of K* values follows standard benchmarks: *K* ≥ 0.74: Excellent agreement beyond chance, *K* between 0.60 and 0.73: Good agreement, *K* between 0.40 and 0.59: Fair agreement, *K* < 0.40: Poor agreement (Wynd et al. 2003).

In this study, both the traditional CVI approach and the modified kappa statistic were utilized to ensure a robust assessment of content validity by adjusting for chance agreement and supporting scale‐level interpretations.

##### 2.6.1.3 Item Analysis

Prior to evaluating face, content, and construct validity, an item analysis was performed to assess the quality and contribution of each item in the FINC‐NA. Given that the scale comprises four distinct subscales—FAM‐RNC, FAM‐CP, FAM‐B, and FAM‐OR—item analysis was conducted separately for each subscale in accordance with COSMIN guidelines for evaluating internal consistency in multidimensional scales (Mokkink et al. 2012).

This process included examining the corrected item–total correlation and Cronbach's alpha if item deleted. The corrected item–total correlation indicates how well each item correlates with the total score of the other items in the same subscale. A threshold of 0.30 or higher was considered acceptable, suggesting adequate item contribution to internal consistency. Items falling below this threshold were carefully reviewed for potential revision or exclusion based on both statistical and conceptual considerations (DeVellis and Thorpe 2021; Tavakol and Dennick 2011).

The second criterion, Cronbach's alpha if item deleted, was used to evaluate whether removing an item would improve the internal consistency of the corresponding subscale. If the alpha value increased upon deletion of an item, this indicated that the item may weaken the scale's reliability. Conversely, if the alpha value decreased after deletion, the item was considered to contribute positively to the internal consistency and was thus retained. However, decisions regarding item removal were not based solely on statistical criteria. Items that slightly reduced reliability were retained if they demonstrated strong content relevance and conceptual alignment with the subscale's intended construct. Additionally, any consideration for item removal required that the item also show a low corrected item–total correlation (typically < 0.30), in order to ensure both psychometric soundness and theoretical coherence. (Nunnally). This approach aligns with established recommendations for evaluating the internal structure of attitude scales using Likert‐type items, particularly in contexts where constructs are multifactorial and theoretically grounded (Mokkink et al. 2012; Tavakol and Dennick 2011).

##### 2.6.1.4 Construct Validity

Construct validity is a fundamental concept in research, especially when using HR‐PRO instruments. It's the degree to which an instrument's scores align with the hypotheses you've made about the construct it's designed to measure. In simpler terms, it's about whether the instrument is truly measuring what it claims to measure.

This validation process involves examining three key areas: 1‐Structural Validity: This refers to the internal relationships of the scores within the instrument. It's about ensuring that the components or items of the instrument are logically connected and form a coherent whole, accurately representing the underlying construct (Tepper and Percy 1994). 2‐Hypothesis Testing: This involves evaluating the instrument's scores against predetermined hypotheses. You might test how the scores from your instrument relate to scores from other, established instruments or how they differ across specific, relevant groups (Viswanathan et al. 2017).

3‐Cross‐Cultural Validity: This aspect focuses on whether the instrument performs consistently across different cultures or linguistic groups. It's essential for ensuring that the tool is not biased and that the observed relationships and differences are meaningful, regardless of the population being studied (Beckstead et al. 2008).

Essentially, assessing construct validity means confirming that an HR‐PRO instrument is a reliable and accurate tool for its intended purpose by examining its internal structure and how its scores behave in relation to other measures and groups (Mokkink et al. 2012).

##### 2.6.1.5 Structural Validity

Prior to conducting the EFA and CFA, preliminary analyses were performed to assess data quality and evaluate statistical assumptions. Given that the sample size exceeded 100 participants, the distribution of each item was examined for univariate normality using skewness and kurtosis indices (Ghasemi and Zahediasl 2012), complemented by visual inspection of histograms and Q–Q plots. Skewness values within ± 3 and kurtosis values within ± 7 were considered indicative of acceptable normality (West et al. 1995). Multivariate normality was assessed using Mardia's coefficient, with values greater than 5 interpreted as evidence of non‐normal distribution (Mardia 1971; Wulandari et al. 2021).

To perform the EFA, all 26 items of the questionnaire were entered as variables into the analysis. Initially, the Kaiser–Meyer–Olkin (KMO) measure of sampling adequacy, Bartlett's test of sphericity, and the Anti‐Image correlation matrix were examined, without applying a minimum correlation threshold for item inclusion. Rotation was initially specified as orthogonal (Varimax). The outputs also included the scree plot and eigenvalues, extracted without imposing a minimum item correlation value (Cases 1–215 in the data set).

Preliminary analyses were performed to assess sample adequacy, determine the optimal number of factors to extract, and evaluate the appropriateness of the rotation method. Bartlett's test of sphericity was used to test the null hypothesis that the correlation matrix is an identity matrix.; a significant result indicated that the data were suitable for factor analysis (Alimohamadi and Sepandi 2019). The Kaiser–Meyer–Olkin (KMO) measure of sampling adequacy was also examined, with values above 0.50 considered acceptable. According to Hutcheson and Sofroniou (1999) (Hutcheson and Sofroniou 1999), KMO values between 0.50 and 0.70 are classified as mediocre, 0.70–0.80 as good, 0.80–0.90 as great, and values above 0.90 as superb. In addition, the Anti‐Image matrix was inspected to evaluate the measure of sampling adequacy (MSA) for each item, with values above 0.50 considered acceptable (Harerimana and Mtshali 2020; Nkansah 2018).

To determine the number of factors to retain, communalities (h^2^ values) were examined for each item, with values between 0.40 and 0.70 considered acceptable for inclusion in the factor solution (Osborne and Costello 2009). The scree plot was visually inspected, and the point of inflection (“elbow”) was used to help determine the optimal number of factors (Zhu and Ghodsi 2006). Furthermore, factors with eigenvalues greater than 1 (Cliff 1988) and explaining more than 5% of the variance (Auerswald and Moshagen 2019) were considered for retention.

In the initial Varimax‐rotated solution, the component matrix revealed complex loadings, with several items loading substantially on multiple factors. This suggested that the underlying constructs were correlated, making an oblique rotation more appropriate (Nguyen and Waller 2023). Therefore, the EFA was re‐run using Principal Component Analysis method with Direct Oblimin rotation. The factor loading matrix was sorted by loading magnitude, and loadings below 0.40 were suppressed in the output. Items with communalities (h^2^) below 0.40 were excluded from the final analysis; these were items 1, 8, and 9.

Regarding CFA was conducted using the maximum likelihood (ML) estimation method to evaluate the factorial validity of the Iranian version of the FINC‐NA questionnaire. Model fit was assessed using multiple fit indices, as recommended in the SEM literature (Kline 2023; Schreiber et al. 2006). The following thresholds were applied to determine acceptable model fit: root mean square error of approximation (RMSEA) < 0.08, standardized root mean square residual (SRMR) < 0.10, normed chi‐square (*X* ^2^/df) < 5, comparative fit index (CFI) > 0.90, Tucker–Lewis index (TLI) > 0.90, Bentler–Bonett normed fit index (NFI) > 0.90, and relative fit index (RFI) > 0.90 (Cases 216–450 in the data set).

##### 2.6.1.6 Hypothesis Testing

The process of hypothesis testing is continuous and iterative in nature. Hypotheses are formulated to represent the expected direction or magnitude of associations and group differences, and each test provides incremental evidence concerning the validity of the construct under investigation. The more consistently empirical findings align with theoretical expectations, the stronger the evidence for construct validity becomes (Mokkink et al. 2012).

In this study, several forms of hypothesis testing were undertaken. First, the hypothesized four‐factor structure of the Iranian version of the FINC‐NA was tested using CFA. Model fit was evaluated by examining multiple fit indices, which are reported in the CFA results section (H1).

Second, convergent validity was assessed through the Average Variance Extracted (AVE), with values above 0.50 considered acceptable, and composite reliability (CR), where values greater than 0.70 were taken as evidence of adequate internal consistency (H2).

Third, discriminant validity was examined using the Maximum Shared Variance (MSV) and in addition to applying the Fornell–Larcker criterion and calculating Hancock's H (MaxR(H)), with values of 0.70 or higher indicating acceptable construct reliability (H3). For these analyses, the Excel macro developed by Dr. James Gaskin (2025 Version) was utilized (Gaskin et al. 2025).

Fourth, known‐groups validity was evaluated by testing hypotheses concerning differences in FINC‐NA total scores across selected demographic characteristics (H4). Specifically, comparisons were conducted based on gender, level of education, the existence of hospital guidelines on family‐centered care, previous experience with family involvement in care, and awareness of the concept of family‐centered care. Independent‐samples *t*‐tests were used for each analysis, with the total FINC‐NA score as the dependent variable and demographic variables as the grouping factor. We considered construct validity adequate if at least 75% of the predefined hypotheses were confirmed.

Finally, ceiling and floor effects were examined by calculating the proportion of respondents who achieved the lowest or highest possible scores on the FINC‐NA. Floor and ceiling effects are indicators of an instrument's sensitivity at the extremes of the scale and may limit interpretability. A threshold of 15% or more of respondents scoring at either extreme was considered indicative of a problematic effect (Gulledge et al. 2019).

##### 2.6.1.7 Responsiveness

Measurement instruments must exhibit a high degree of sensitivity to detect and accurately capture changes, as well as sufficient responsiveness to promptly reflect these changes. According to the COSMIN checklist, responsiveness is defined as the ability of a HR‐PRO instrument to detect meaningful changes in the construct of interest over time (Mokkink et al. 2012). Terwee et al. (2007) propose that responsiveness can be evaluated by comparing the smallest detectable change (SDC) with the minimally important change (MIC). An instrument is considered responsive when the SDC is smaller than the MIC.

In the present study, the calculation of responsiveness parameters was conducted manually following the distribution‐based approach for the MIC. Initially, the standard error of measurement (SEM) was computed using the formula:

SEM=SD×√((1)−ICC)

Subsequently, the SDC at the individual level was calculated as:

$${\text{SDC}}_{\text{Individual}}=1.96\times \surd 2\times SEM$$

The pooled standard deviation (SD) was then determined using the following formula:

$${\text{SD}}_{\text{Pooled}}=\sqrt{\frac{{SD}_{T1}^{2}+{SD}_{T2}^{2}}{2}}$$

Finally, the MIC was estimated based on the distribution‐based method:

$$\text{MIC}=0.5\times {SD}_{Pooled}$$

These calculations allowed for the assessment of the instrument's ability to detect meaningful changes over time.

##### 2.6.1.8 Interpretability

Interpretability refers to the extent to which the scores obtained from a measurement instrument can be meaningfully understood and translated into practical or clinical terms (Mokkink et al. 2012; Stinson et al. 2006). In this study, interpretability was evaluated by examining the SEM, the SDC, and the MIC. The SEM provides information on the precision of individual measurements (Harvill 1991), while the SDC represents the minimal change that exceeds measurement error (van Kampen et al. 2013). The MIC indicates the smallest change considered meaningful or important for participants (Cliff 1988).

By comparing the SDC and MIC values, it is possible to determine whether meaningful changes can be detected at the individual level or, if not, whether the instrument remains useful for detecting changes at the group level. These parameters were calculated manually using the distribution‐based method, allowing for a clear understanding of the instrument's capacity to produce interpretable scores and to identify clinically relevant changes over time.

#### 2.6.2 Reliability

Reliability reflects the degree to which a measurement is free from error and produces stable and consistent results. Its assessment commonly involves examining three main aspects: internal consistency, test–retest reliability, and measurement error (Mokkink et al. 2012).

##### 2.6.2.1 Internal Consistency

Internal consistency reflects the degree to which items within a scale are interrelated and collectively measure the same underlying construct (Mokkink et al. 2012). In this study, the internal consistency of the overall instrument and its six subscales was examined using Cronbach's alpha and McDonald's omega coefficients. For the interpretation of reliability coefficients, commonly accepted thresholds were applied. Values above 0.90 were considered excellent, 0.80 to 0.89 good, and 0.70 to 0.79 acceptable. Coefficients between 0.60 and 0.69 were regarded as questionable, 0.50 to 0.59 as poor, and values below 0.50 as unacceptable (Taber 2018; Terwee et al. 2007). These criteria were used for both Cronbach's alpha and McDonald's omega to evaluate the internal consistency of the instrument and its subscales.

##### 2.6.2.2 Test‐Retest Reliability

Test–retest reliability reflects the degree to which an instrument yields consistent results when administered to individuals whose health status remains stable over time (Mokkink et al. 2012). In line with the COSMIN guidelines, the test–retest procedure in this study was conducted with a 2‐week interval. This time frame was selected to minimize the likelihood of participants recalling their previous responses, while also allowing for the possibility of natural fluctuations in health status (Mokkink et al. 2012).

To evaluate stability, the questionnaire was administered to a sample of 20 nurses on two separate occasions, with a 14‐day interval between administrations. The obtained scores were then analyzed using the intraclass correlation coefficient (ICC). For interpretation, ICC values below 0.50 were regarded as indicative of poor reliability, values between 0.50 and 0.75 as moderate reliability, values from 0.75 to 0.90 as good reliability, and values above 0.90 as excellent reliability (Koo and Li 2016).

##### 2.6.2.3 Measurement Error

Measurement error is a central indicator of reliability, reflecting both systematic and random errors in scores that are not due to true changes in the construct being assessed (Terwee et al. 2007). In this study, measurement error was evaluated using the SEM and the SDC. The SEM represents the precision of individual scores, while the SDC indicates the minimum change required to be interpreted as a real change rather than error. Lower SDC values correspond to higher measurement sensitivity (Terwee et al. 2007).

### 2.7 Statistical Analysis

All statistical analyses were performed using SPSS software, version 27 and AMOS, version 24. Descriptive statistics were applied to summarize sociodemographic characteristics. Categorical variables were presented as frequencies and percentages, while continuous variables were described using minimum and maximum values, as well as mean and standard deviation (SD).

### 2.8 Patient or Public Contribution

Nurses participated voluntarily in completing the Persian version of the FINC‐NA and the test–retest phase. They provided feedback on item clarity and cultural relevance during the face validity assessment. Although patients or family members were not directly involved in the study design or data interpretation, nurses' perspectives offered valuable insights into the practical and cultural aspects of family‐centered nursing care within Iranian clinical settings.

## 3 Results

### 3.1 Descriptive and Hospital Related Characteristics of Nurses

Participants in the EFA and CFA groups showed comparable socio‐demographic and occupational characteristics, with a mean age of approximately 34.5 years and similar average work experience (10.0 vs. 10.8 years, respectively). In both groups, the majority were female, held a bachelor's degree, were married, and were employed as civil service nurses.

The sample included nurses from various clinical wards, with internal medicine and surgical units being the most represented. A considerable proportion of participants reported working extended hours and rotating shifts, while approximately 15% were employed in more than one hospital. Around 61% of participants held professional nursing qualifications. Less than half reported the existence of institutional guidelines on family involvement in care, and awareness of such guidelines was even lower. Slightly more than half reported considering family involvement in care and having prior experience engaging families in care, whereas only a small proportion had received formal education in this area. In addition, more than half reported having a family member with prior hospitalization experience, and approximately half to two‐thirds were familiar with the concept of family‐centered care (Table 1).

**TABLE 1:** Socio‐demographic and hospital‐related characteristics of participants in the EFA and CFA groups (*N* = 430).

Characteristics		Category	EFA (*n* = 215)	CFA (*n* = 215)
Continuous variable	Age (years)	—	(34.4 ± 7.4) 22–53	(34.7 ± 7.6) 23–55
	Work experience (Year)	—	(10.0 ± 6.9) 1–29	(10.8 ± 7.6) 1–30
Categorical variable	Gender	Male	91 (42.3)	85 (39.5)
		Female	123 (57.2)	125 (58.1)
	Education level	Bachelor	190 (88.4)	192 (89.3)
		Master's degree or higher	21 (9.8)	22 (10.2)
	Marital status	Married	141 (65.6)	124 (57.7)
		Single	67 (31.2)	83 (38.6)
		Divorce or widow	6 (2.8)	4 (1.9)
	Employment status	Mandatory service nurses	47 (21.9)	37 (17.2)
		Long term contract nurses	19 (8.8)	17 (7.9)
		Civil service nurses	111 (51.6)	106 (49.3)
		Agency/Contractual nurses	34 (15.8)	46 (21.4)
	Clinical wards	Internal medicine	65 (30.2)	26 (12.1)
		Surgical	41 (19.1)	71 (33.0)
		Intensive care	46 (21.4)	59 (27.4)
		Emergency	32 (14.9)	23 (10.7)
		Mental	26 (12.1)	31 (14.4)
	Weekly working	Standard	69 (32.1)	90 (41.9)
		Excessive	137 (63.7)	120 (55.8)
	Shift pattern	Fixed	45 (20.9)	47 (21.9)
		Rotating	169 (78.6)	164 (76.3)
	Employment at multiple hospitals	Yes	33 (15.3)	31 (14.4)
		No	181 (84.2)	184 (85.6)
	Professional qualification held	Yes	132 (61.4)	134 (62.3)
		No	83 (38.6)	81 (37.7)
	Hospital guidelines on family involvement	Have	66 (30.7)	97 (45.1)
		Do not have	147 (68.4)	118 (54.9)
	Awareness of hospital guidelines	Yes	62 (28.8)	98 (45.6)
		No	135 (62.8)	117 (54.4)
	Consideration of family involvement in care	Yes	103 (47.9)	120 (55.8)
		No	111 (51.6)	95 (44.2)
	Previous experience with family engagement in care	Yes	119 (55.3)	115 (53.5)
		No	94 (43.7)	88 (40.9)
	Participated in education regarding family involvement	Yes	32 (14.9)	55 (25.6)
		No	180 (83.7)	150 (69.8)
	Has a family member experienced an illness requiring professional care?	Yes	119 (55.3)	140 (65.1)
		No	93 (43.3)	65 (30.2)
	Familiarity with the concept of family‐centered care	Yes	111 (51.6)	137 (63.7)
		No	102 (47.4)	68 (31.6)

*Note:* Values are presented as mean ± standard deviation (range) or *n* (%).

Abbreviations: agency/contractual nurses = nurses employed through private agencies or contractual arrangements; CFA = confirmatory factor analysis; civil service nurses = government‐employed nurses; EFA = exploratory factor analysis; long‐term contract nurses = nurses employed under renewable long‐term contracts; Mandatory service nurses = nurses completing the national compulsory service program; SD = standard deviation.

### 3.2 Validity

The Persian version of the FINC‐NA demonstrated strong evidence of content and face validity. CVR values ranged from 0.57 to 1.00, with a mean CVR of 0.93, exceeding the acceptable threshold for a 12‐member expert panel. Although three items (11, 17, and 22) showed comparatively lower CVR values, all remained above the minimum acceptable level.

Across clarity, simplicity, and relevance domains, most items achieved I‐CVI values of 1.00, indicating strong expert agreement on item quality. Only a few items (e.g., 17 and 24) demonstrated slight reductions in clarity or simplicity ratings, although these remained acceptable. At the scale level, S‐CVI/Ave and S‐CVI/UA values confirmed excellent content validity across all three domains.

Face validity was also strongly supported, as all items exceeded the acceptable impact score threshold of 1.5, with values ranging from 2.87 to 4.40, indicating that items were considered important and relevant by the target population. In addition, modified kappa values for selected items indicated excellent agreement beyond chance. These findings collectively support the adequacy of content and face validity for the Persian FINC‐NA (Table 2).

**TABLE 2:** Content and face validity indices for the Persian version of the FINC‐NA (*N* = 12 experts and 10 nurses).

Item	CVR (Necessity)	I‐CVI (Clarity)	I‐CVI (Simplicity)	I‐CVI (Relevance)	Impact score	K*
Item 1	0.85	1	1	1	3.32	*
Item 2	0.85	1	1	1	3.16	*
Item 3	1	1	1	1	4.22	*
Item 4	1	1	1	1	4.4	*
Item 5	1	1	1	1	4.3	*
Item 6	1	1	1	1	4.08	*
Item 7	1	1	1	1	4.15	*
Item 8	1	1	1	1	3.73	*
Item 9	1	1	1	1	4.08	*
Item 10	1	1	1	1	3.48	*
Item 11	0.57	1	1	1	4.35	*
Item 12	0.85	1	1	1	4.25	*
Item 13	1	1	1	1	3.69	*
Item 14	0.85	1	1	1	4.03	*
Item 15	1	1	1	0.92	3.78	0.9
Item 16	1	1	1	1	3.04	*
Item 17	0.71	1	0.85	0.92	3.69	0.91
Item 18	1	1	1	1	3.87	*
Item 19	1	1	1	1	4.275	*
Item 20	1	1	1	1	4.13	*
Item 21	1	1	1	1	4.13	*
Item 22	0.71	0.85	0.92	1	2.88	*
Item 23	1	1	1	1	3.82	*
Item 24	1	0.85	0.85	0.92	4.13	0.91
Item 25	1	1	1	1	3.99	*
Item 26	1	1	1	1	2.87	*

*Note:* Mean CVR for scale = 0.93, For Clarity domain (S‐CVI/UA = 0.92, S‐CVI/Ave/Item = 0.98), For simplicity domain (S‐CVI/UA = 0.88, S‐CVI/Ave/Item = 0.98), For relevance domain (S‐CVI/UA = 0.88, S‐CVI/Ave/Item = 0.99). *K* was not calculated for items with I‐CVI = 1.00 because perfect agreement was observed among experts.

Abbreviations: CVR = Content Validity Ratio; I‐CVI = Item‐level Content Validity Index; K* = modified Kappa coefficient; S‐CVI/Ave = Scale‐level Content Validity Index based on the Average method; S‐CVI/UA = Scale‐level Content Validity Index based on Universal Agreement.

Item‐level psychometric evaluation was conducted for the four subscales (Fam‐RNC, Fam‐CP, Fam‐B, and Fam‐OR). Across all subscales, corrected item–total correlations exceeded 0.30, and no improvement in Cronbach's alpha was observed following item deletion, supporting item retention and internal consistency (Table 3). These results indicate strong internal consistency and support retaining all items in this subscale. Overall, no items fell below the 0.30 threshold for corrected item–total correlation, and none met both statistical and theoretical criteria for deletion. Thus, all items were retained across the four subscales, and the scale demonstrated acceptable internal consistency in accordance with COSMIN guidelines and established psychometric standards. Lastly, it should be noted that the above item analysis was conducted according to the original four subscales of the FINC‐NA as defined in the source instrument. However, subsequent exploratory and confirmatory factor analyses in the present study yielded a revised factor structure more suited to the cultural and linguistic context. The structural validity results presented in the following section are therefore based on this empirically derived factor solution.

**TABLE 3:** Item analysis of the Persian version of the FINC‐NA according to the original subscale structure (*N* = 430).

Subscale	Item	Corrected item–Total correlation	Cronbach's alpha if item deleted
Fam‐RNC (*α* = 0.86)	Item 3	0.50	0.85
	Item 4	0.58	0.84
	Item 5	0.63	0.84
	Item 7	0.59	0.84
	Item 10	0.47	0.85
	Item 11	0.66	0.84
	Item 13	0.50	0.85
	Item 20	0.62	0.84
	Item 21	0.57	0.85
	Item 22	0.56	0.85
Fam‐CP (*α* = 0.83)	Item 1	0.38	0.83
	Item 6	0.59	0.81
	Item 9	0.40	0.83
	Item 12	0.55	0.81
	Item 14	0.61	0.80
	Item 15	0.65	0.80
	Item 19	0.65	0.80
	Item 24	0.65	0.80
Fam‐B (*α* = 0.58)	Item 2	0.34	0.53
	Item 8	0.30	0.56
	Item 23	0.41	0.47
	Item 26	0.41	0.47
Fam‐OR (*α* = 0.77)	Item 16	0.63	0.70
	Item 17	0.57	0.72
	Item 18	0.59	0.72
	Item 25	0.54	0.74

*Note:* Corrected item–total correlations ≥ 0.30 were considered acceptable.

Abbreviations: Fam‐B = Family as a Burden; Fam‐CP = Family as a Conversational Partner; Fam‐OR = Family as Its Own Resource; Fam‐RNC = Family as a Resource in Nursing Care.

All items showed skewness within ± 3 and kurtosis within ± 7, indicating acceptable univariate normality. Mardia's coefficient was below 8, confirming multivariate normality.

Missing data were assessed at the item level for all questionnaires during data collection and data entry. To minimize missing responses, questionnaires were completed in the presence of the researchers, and each form was checked immediately after completion. In cases of incomplete responses, participants were politely asked to complete the missing items at the time of data collection. During data screening, no missing data were identified.

Multivariate outliers were examined using Mahalanobis distance (D^2^), with a significance level of *p* < 0.001 considered indicative of potential outliers (Ghorbani 2019; McLachlan 1999). The analysis indicated no multivariate outliers in the dataset.

**FIGURE 1:**
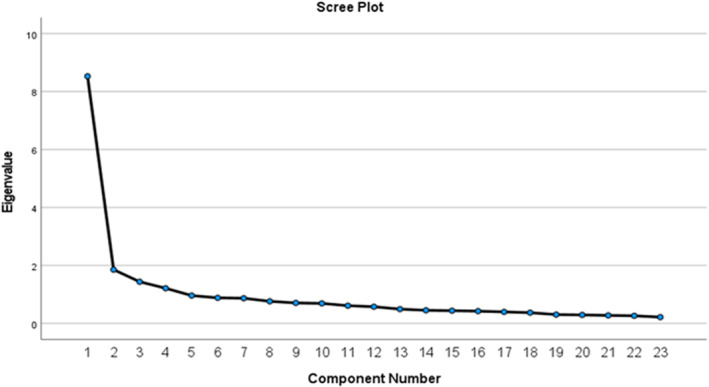
Factor load scree plot of the item for determining the number of extracted factors of the Iranian version of FINC‐NA.

Visual inspection of the scree plot revealed a clear inflection point (“elbow”) at the fourth factor, indicating that a four‐factor solution was the most appropriate for the Iranian version of the FINC‐NA. This was consistent with the eigenvalue criterion (> 1) and the theoretical structure of the instrument (Figure 1).

The final EFA, using Principal Component Analysis with Direct Oblimin rotation, yielded a four‐factor solution explaining 56.65% of the total variance (Table 4). Factor 1 accounted for 37.08% of the variance and included 12 items primarily reflecting the family's active participation in care provision, joint decision‐making, and involvement in healthcare processes. Based on the theoretical framework of family involvement in nursing care, this factor was named Family as an Active Care Partner (Fam‐ACP). Factor 2, comprising three items and explaining 8.06% of the variance, reflected emotional encouragement, motivation, and perceived support provided by the family during the care process. In line with these themes, this factor was named Family Engagement and Support (Fam‐ES). Factor 3 consisted of two items contributing 6.24% of the variance, which focused on the emotional and practical strain perceived by families in the caregiving role. This factor was therefore named Family Burden (Fam‐B). Factor 4, with six items accounting for 5.27% of the variance, merged concepts originally captured by the “Family as its Own Resource” and “Family as a Conversational Partner” subscales in the original instrument. The items in this factor emphasized the family's internal coping capacity, problem‐solving abilities, and communicative role in interactions with healthcare providers. Based on these overlapping themes, this factor was named Family Strengths and Communication (Fam‐SC).

**TABLE 4:** Factor analysis of the Iranian version of FINC‐NA based on EFA (*n* = 215).

Scale item	Factors				Anti‐image diagonal (MSA)	h^2^
	1	2	3	4		
Item 1 (omitted in our study)					—	—
Item 2		0.636			0.615	0.501
Item 3				0.760	0.930	0.589
Item 4				0.809	0.919	0.652
Item 5				0.691	0.891	0.668
Item 6				0.669	0.946	0.601
Item 7		0.448			0.890	0.591
Item 8 (omitted in our study)					—	—
Item 9 (omitted in our study)					—	—
Item 10		0.638			0.804	0.628
Item 11				0.533	0.880	0.546
Item 12				0.458	0.911	0.496
Item 13	0.619				0.887	0.400
Item 14	0.595				0.921	0.550
Item 15	0.524				0.961	0.557
Item 16	0.635				0.931	0.547
Item 17	0.636				0.901	0.515
Item 18	0.553				0.904	0.570
Item 19	0.753				0.925	0.569
Item 20	0.806				0.891	0.593
Item 21	0.612				0.928	0.518
Item 22	0.663				0.931	0.596
Item 23			0.743		0.619	0.598
Item 24	0.675				0.938	0.573
Item 25	0.647				0.889	0.473
Item 26			0.828		0.437	0.676
% of variance observed	37.08	8.06	6.24	5.27	Total Score = 56.65	
Cumulative %	37.08	45.15	51.39	56.67		

*Note:* Only factor loadings ≥ 0.40 are displayed. Items 1, 8, and 9 were removed because of low communalities (< 0.40). KMO = 0.903. Bartlett's test of sphericity: χ^2^(253) = 2207.09, *p* < 0.001. The four factors were labeled Family as an Active Care Partner (Fam‐ACP), Family Engagement and Support (Fam‐ES), Family Burden (Fam‐B), and Family Strengths and Communication (Fam‐SC).urden (Fam‐B), Factor 4 = Family‐Strengths and Communication (Fam‐SC).

Abbreviations: EFA = exploratory factor analysis; *h* ^2^ = communality; MSA = measure of sampling adequacy.

All retained items demonstrated acceptable communalities (*h* ^2^ ≥ 0.40) and factor loadings ≥ 0.40. Items 1, 8, and 9 were excluded due to low communalities. The sampling adequacy for the analysis was confirmed by a KMO value of 0.903, and Bartlett's test of sphericity was significant (*X* ^*2*^ = 2207.09, df = 253, *p* < 0.001), indicating that the data were suitable for factor analysis.

**FIGURE 2:**
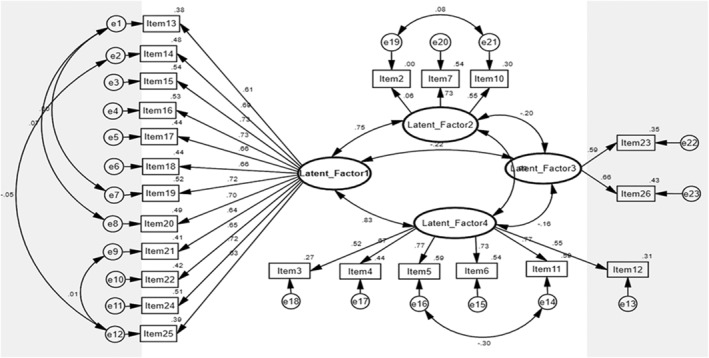
Factor structure model of the Iranian version of FINC‐NA based on CFA. Latent‐factor 1:Family as an Active Care Partner (Fam‐ACP), Latent‐Factor 2 = Family‐Engagement and Support (Fam‐ES), Latent‐Factor 3 = Family‐burden (Fam‐B), Latent‐Factor 4 = Family‐Strengths and Communication (Fam‐SC).

The four‐factor model of the Iranian version of the FINC‐NA, identified through EFA, was tested using CFA (Figure 2). The model demonstrated an acceptable fit to the data: *X* ^*2*^ (218) = 790.6, *χ* ^2^/df = 3.63 (< 5), *p* < 0.001, CFI = 0.901, TLI = 0.949, SRMR = 0.069 (< 0.10), RMSEA = 0.078 (< 0.08), NFI = 0.910, and RFI = 0.932, all within recommended thresholds for acceptable model fit.

Standardized factor loadings for all items were statistically significant (*p* < 0.001) and ranged from 0.27 to 0.83. Factor 1 (Family as an Active Care Partner, Fam‐ACP) included 12 items with loadings between 0.39 and 0.73. Factor 2 (Family Engagement and Support, Fam‐ES) comprised three items with loadings of 0.54–0.73. Factor 3 (Family Burden, Fam‐B) included two items with loadings of 0.59 and 0.66. Factor 4 (Family Strengths and Communication, Fam‐SC) contained six items with loadings from 0.27 to 0.77 (Table 5).

**TABLE 5:** Confirmatory factor analysis parameter estimates for the Persian version of the FINC‐NA (*n* = 215).

Subscale	Item	Estimate	Standard error	*p*‐value
Family as an active care partner (Fam‐ACP)	Item 13	1	Refference category	
	Item 14	1.273	0.108	< 0.001
	Item 15	1.238	0.100	< 0.001
	Item 16	1.341	0.109	< 0.001
	Item 17	1.137	0.099	< 0.001
	Item 18	1.254	0.109	< 0.001
	Item 19	1.227	0.100	< 0.001
	Item 20	1.177	0.095	< 0.001
	Item 21	1.079	0.096	< 0.001
	Item 22	1.198	0.106	< 0.001
	Item 24	1.284	0.105	< 0.001
	Item 25	1.049	0.096	< 0.001
Family‐strengths and communication (Fam‐SC).	Item 12	1	Refference category	
	Item 11	1.527	0.136	< 0.001
	Item 6	1.383	0.125	< 0.001
	Item 5	1.590	0.142	< 0.001
	Item 4	1.310	0.126	< 0.001
	Item 3	1.002	0.114	< 0.001
Family‐engagement and support (Fam‐ES)	Item 2	1	Refference category	
	Item 7	1.002	0.114	< 0.001
	Item 10	1.341	0.109	< 0.001
Family‐burden (Fam‐B)	Item 23	1	Refference category	
	Item 26	1.110	0.446	< 0.001

*Note:* The model fit indicators: *X* ^2^ = 790.6, Degree of freedom = 218, *X* ^2^/df = 3.627 (Acceptable value: < 5), *p*‐value < 0.001 (Acceptable < 0.05), CFI = 0.901 (Acceptable value: > 0.900), TLI = 0.949 (Acceptable value: > 0.90), SRMR = 0.069 (Acceptable value: < 0.10), RMSEA = 0.078 (Acceptable value: < 0.08), NFI = 0.910 (Acceptable value: > 0.90), RFI = 0.932 (Acceptable value: > 0.90).

All regression weights were significant (*p* < 0.001), supporting the convergent validity of the items within each factor. The factor structure was consistent with the theoretically driven reclassification from the EFA, confirming the multidimensional construct of family involvement in nursing care within the Iranian context.

In addition, several correlations between error terms (measurement residuals) were specified to improve model fit, consistent with the modification indices and theoretical considerations. For example, error terms of Item13 (e1) with Item19 & Item 20 (e7 & e8), Item14 (e2) with Item25 (e12), and Item21 (e9) with Item25 (e12), Item2 (e19) with Item10 (e21), and Item 5 (e16) with Item11 (e14) were allowed to correlate, reflecting shared variance not explained by the latent factors. These correlations suggest some overlap in item wording or content that is conceptually meaningful within the Iranian cultural adaptation.

#### 3.2.1 Hypothesis Testing, Responsiveness and Interpretability

Composite reliability values exceeded the recommended threshold of 0.70 for the FINC‐ACP (CR = 0.83) and FINC‐ES (CR = 0.91) subscales, indicating acceptable reliability for these dimensions. In contrast, the FINC‐B (CR = 0.45) and FINC‐SC (CR = 0.56) subscales did not meet the recommended cutoff, suggesting weaker internal consistency. With respect to convergent validity, AVE values ranged from 0.28 to 0.46. Although none of the factors reached the recommended 0.50 threshold, both FINC‐ACP and FINC‐ES fell within the acceptable range of 0.36–0.50. Discriminant validity was not fully supported, as the MSV exceeded the AVE for several subscales. Construct reliability using Hancock's H, which showed adequate values for FINC‐ACP (0.84) and FINC‐ES (0.91), but lower values for FINC‐B (0.61) and FINC‐SC (0.56) (Table 6).

**TABLE 6:** Scale subscale score, stability coefficients, inter class correlation coefficients of the Iranian version of FINC‐NA (*n* = 430).

Factors	Cronbach's *α* coefficient	McDonald's omega	ICC (95% CI)^a^	SEM	SDC	MIC	CR	AVE	MSV	MaxR(H)	Floor effect (%)	Ceiling effect (%)
FINC‐ACP	0.911	0.910	0.839 (0.600–0.936)	3.821	10.55	4.88	0.83	0.45	0.81	0.84	0.46	0.46
FINC‐ES	0.571	0.571	0.158 (−0.095–0.485)	1.465	4.03	0.93	0.91	0.46	0.68	0.91	2.09	0.46
FINC‐B	0.614	—^b^	0.536 (0.088–0.794)	1.502	4.14	1.18	0.45	0.28	0.81	0.61	2.79	4.88
FINC‐SC	0.853	0.852	0.632 (0.120–0.851)	2.663	7.35	2.55	0.56	0.39	0.04	0.56	0.69	3.72

Abbreviations: AVE = Average Variance Extracted (accepted if AVE > 0.5, the threshold is 0.36–0.5; regarding convergent validity); CI = Confidence Intervale; CR = Composite Reliability (CR > 0.7, regarding reliability); ICC = Inter Class correlation Coefficient; MaxR(H) = Maximum Reliability H or Hancock's H (accepted if ≥ 0.70 indicate acceptable construct reliability); MIC = Minimal Important Change; MSV = Maximum Shared Square Variance (accepted if MSV < AVE; regarding discriminant validity); SDC = Smallest Detectable Change; SEM = Standard Error of the Measurement.

^a^ all reported parameters are statistically significant.

^b^ Omega cannot be estimated because the number of items is less than 3.

Floor and ceiling effects were also evaluated. Across all subscales, the proportion of participants achieving either the minimum or maximum possible scores was well below the 15% threshold, indicating that floor and ceiling effects were not a concern in this study. The highest floor effect was observed for FINC‐B at 2.79% and the highest ceiling effect was also in FINC‐B at 4.88%.

Known‐groups validity was examined by testing differences in the total FINC‐NA score across selected demographic variables. No significant difference was observed between male and female nurses (*p* = 0.061). However, significant differences emerged in other comparisons. Nurses working in hospitals with family‐centered care guidelines reported more positive attitudes toward family involvement in care (*p* < 0.001). Similarly, nurses with previous experience involving families in care and those who had prior awareness of the concept of family‐centered care scored significantly higher on the FINC‐NA total scale (*p* < 0.001 for both comparisons). Finally, nurses with a master's degree or higher also demonstrated significantly more positive attitudes compared with those holding only a bachelor's degree (*p* = 0.013). Overall, four out of the five predefined hypotheses were confirmed, representing 80% confirmation and thereby supporting adequate construct validity according to COSMIN recommendations.

The MIC represents the smallest change in a measured parameter that is considered clinically or practically meaningful. In this study, the MIC values for the FINC subscales ranged from 0.93 to 4.88 units, whereas the SDC values ranged from 4.03 to 10.55 units. Across all subscales, the MIC values were smaller than the corresponding SDC values, indicating that the instrument may not reliably detect individual‐level changes that are considered meaningful.

The SEM values, ranging from 1.47 to 3.82 units, suggest that the instrument exhibits an acceptable level of precision. Although the SDC exceeds the MIC, the instrument can still detect meaningful changes at the group level, supporting its responsiveness for use in research or clinical settings where mean changes across groups are evaluated. Overall, these findings indicate that the Iranian version of the FINC scale is sufficiently precise and capable of detecting relevant changes in the measured constructs at the population level, while caution should be exercised when interpreting changes at the individual level.

Regarding interpretability results showed that across all subscales, the MIC values were smaller than the corresponding SDC values, suggesting that the instrument may not reliably detect meaningful changes at the individual level. However, these values indicate that the FINC scale is suitable for detecting meaningful changes at the group level, where averaging across participants reduces measurement error. Overall, the combination of SEM, SDC, and MIC values provides a clear understanding of the scale's interpretability and its capacity to generate meaningful and actionable scores within research and clinical contexts.

### 3.3 Reliability

The internal consistency of the instrument was examined using Cronbach's alpha and McDonald's omega coefficients. The overall FINC‐ACP and FINC‐SC subscales demonstrated strong internal consistency, with coefficients exceeding 0.85. In contrast, the FINC‐ES and FINC‐B subscales yielded lower values, ranging from 0.57 to 0.61, which may be interpreted as moderate reliability in exploratory research contexts.

Test–retest reliability, assessed with ICC, varied across subscales. The FINC‐ACP subscale achieved an ICC of 0.839 (95% CI: 0.600–0.936), indicating good stability over time. The FINC‐SC and FINC‐B subscales showed moderate reliability, with ICCs of 0.632 (95% CI: 0.120–0.851) and 0.536 (95% CI: 0.088–0.794), respectively. By contrast, the FINC‐ES subscale demonstrated poor test–retest reliability, with an ICC of 0.158 (95% CI: −0.095–0.485).

Measurement error was further evaluated using the SEM and the SDC. The SEM values ranged from 1.47 to 3.82, while the SDC values ranged from 4.03 to 10.55. These results indicate that the instrument exhibits satisfactory precision overall, although sensitivity to detect true change varies between subscales. Finally, it should be noted that the new scoring system is provided in Supporting Information S2: Section file 2.

## 4 Discussion

The purpose of this study was to examine the psychometric properties of the FINC‐NA questionnaire among Iranian nurses, using the COSMIN checklist as a methodological guide. The results showed that the Persian version of the FINC‐NA has acceptable levels of validity, reliability, responsiveness, and interpretability when used in Iranian healthcare settings. The FINC‐NA was originally developed to measure nurses' attitudes toward the role of families in the care process and is grounded in a multidimensional understanding of family involvement in nursing (Benzein et al. 2008). Although several instruments exist to assess nurse–family relationships or the perceived contribution of families to patient care, many focus narrowly on domains such as communication or caregiver burden, and do not capture the full spectrum of attitudes (e.g., including perceived risk, stigma, fear). For instance, a recent COSMIN‐based review of caregiver instruments found that most tools measure burden or distress, but omit domains like responsiveness or risk perception (Zhou et al. 2025). The FINC‐NA is more comprehensive, as it evaluates multiple domains including the family as a resource in nursing care, family as a conversational partner, family as a burden, and collaboration with family members. Assessing this tool within the Iranian cultural context is particularly valuable, since family involvement is a central component of patient care and has a strong influence on both care outcomes and nurses' professional attitudes.

In the content and face validity stages, items were judged relevant and important by an expert panel and by nurses in the target population (mean CVR = 0.93; item impact scores > 1.5), indicating that the translation process achieved conceptual and linguistic equivalence with the original instrument. These results are consistent with recommended cross‐cultural procedures and mirror the rigorous translation steps described for other FINC‐NA adaptations (Benzein et al. 2008).

Before conducting EFA, the adequacy of the data was verified. The KMO measure of sampling adequacy was 0.908, indicating that the data were suitable for factor analysis, and Bartlett's test of sphericity was significant (*χ* ^2^ = 4583.214, *p* < 0.001), confirming that correlations among items were sufficiently large to justify factor extraction. The EFA revealed a four‐factor solution, consisting of Family as an Active Care Partner (Fam‐ACP), Family Engagement and Support (Fam‐ES), Family Burden (Fam‐B), and Family Strengths and Communication (Fam‐SC), which together explained 56.65% of the total variance. Three items from the original FINC‐NA (items 1, 8, and 9) were excluded because of low communalities and weak factor loadings. The factorial structure was then tested in an independent CFA sample, and the model demonstrated acceptable fit indices (*χ* ^2^/df = 3.63, CFI = 0.901, TLI = 0.949, RMSEA = 0.078), supporting the construct validity of the Persian version in the Iranian nursing population.

The findings of the present study are largely consistent with international research on the FINC‐NA while also reflecting specific cultural adaptations required in the Iranian context. Similar to the original Swedish version, the Persian FINC‐NA yielded a four‐factor solution that explained a comparable proportion of variance (56.65%), indicating that the fundamental multidimensional structure of the instrument was preserved (Benzein et al. 2008). The Spanish validation also confirmed four factors with satisfactory reliability (Cronbach's *α* = 0.769–0.888) and explained 54% of the variance (Pascual Fernández et al. 2015). The European Portuguese version demonstrated high internal consistency (*α* = 0.87) and supported the same factorial model (Oliveira Pda et al. 2011). The Dutch study using Rasch modeling likewise upheld the original four‐subscale structure, confirming the robustness of the FINC‐NA across cultural settings (Hagedoorn et al. 2018). In contrast, the Brazilian adaptation proposed a three‐factor, 18‐item model after removing items with weak loadings, and the German version retained four dimensions but required the elimination of seven items to improve model fit (Naef et al. 2021; Ruiz et al. 2022). Taken together, these results suggest that while the FINC‐NA maintains a stable core structure across diverse societies, certain modifications in item composition or subscale interpretation are often needed to accommodate contextual and cultural differences. The present Persian version therefore contributes further evidence to the cross‐cultural applicability of the FINC‐NA, supporting its theoretical foundation and demonstrating its relevance for assessing nurses' attitudes toward family involvement within the sociocultural framework of Iranian healthcare.

Internal consistency and test–retest reliability varied across the subscales of the Persian FINC‐NA. The Fam‐ACP subscale demonstrated excellent internal consistency (Cronbach's *α* = 0.91; McDonald's *ω* = 0.91) and strong temporal stability (ICC = 0.839), indicating that this dimension was well represented and conceptually coherent among Iranian nurses. Similarly, the Fam‐SC subscale showed good internal consistency (*α* = 0.85) and acceptable test–retest reliability (ICC = 0.63). However, the Fam‐ES and Fam‐B subscales performed less well, with Cronbach's *α* values of 0.57 and 0.61 and ICCs of 0.158 and 0.54, respectively. Comparable patterns have been observed in other cultural adaptations. For example (Naef et al. 2021), reported lower reliability for the burden subscale in the German FINC‐NA, and (Pascual Fernández et al. 2015) found that several items within the Spanish version had weaker item–total correlations, requiring refinement. These findings suggest that certain items may not resonate equally across settings, possibly due to differences in how emotional support, dependency, or family burden are interpreted in various healthcare cultures (Chomeya and Piyakun 2022; Tsai et al. 2025). In the Iranian context, where family presence is often considered a moral duty and a natural component of care, nurses may perceive less variation in the “burden” or “support” domains, which could reduce internal consistency. Limited item numbers in these subscales may have further contributed to the lower reliability indices.

Convergent and discriminant validity in our study also revealed cultural nuances. The AVE values in our data fell short of the conventional 0.50 threshold (0.28–0.46), indicating that, in each factor, items shared less variance than ideal. The Brazilian adaptation similarly dropped items with weak loadings and restructured the instrument into three factors to enhance conceptual clarity, rather than retaining the original four domains intact (Ruiz et al. 2022). In contrast, the Dutch version preserved four subscales using Rasch modeling and reported that most items showed good discrimination and monotonic behavior (Hagedoorn et al. 2018). In our sample, overlap was also detected, as MSV exceeded AVE in certain dimensions, suggesting that Iranian nurses' attitudes toward family participation may not be sharply differentiated across constructs. This may reflect cultural interdependence and blurred boundaries between caring roles in Iranian nursing practice. Given the limited reporting of discriminant validity statistics in many adaptations, future translation studies should include AVE/MSV analyses and cognitive interviewing to explore how nurses interpret overlapping family‐attitude concepts in different cultural settings.

From the perspective of responsiveness and interpretability, the Persian FINC‐NA demonstrated sufficient measurement precision but limited sensitivity to small individual‐level changes. In our study, the MIC values were smaller than the SDC across all subscales, indicating that meaningful changes at the individual level may be obscured by measurement error. The relatively small size of the test–retest sample (*n* = 20) further reduces the precision of reliability and responsiveness estimates.

Several factors may account for the relatively lower or borderline psychometric indices observed in certain subscales. Conceptual differences in how Iranian nurses view family involvement, combined with translation nuances and cultural connotations, may have influenced item interpretation. For example, expressions reflecting “burden” or “support” could be perceived differently in collectivist societies, where family engagement is seen as a moral norm rather than an optional component of care. Moreover, slight reconfiguration of item loadings during factor analysis, necessary to achieve adequate model fit, may reflect underlying cultural distinctions in nurse–family dynamics. Similar item reallocation has been reported in other adaptations, including the Spanish and German versions, suggesting that the FINC‐NA is sensitive to contextual variations in the meaning of family participation.

The findings suggest that the Persian FINC‐NA can effectively capture nurses' attitudes toward family involvement in clinical care, providing a culturally relevant tool for both research and practice. Understanding these attitudes is crucial for designing interventions that promote family‐centered care and improve patient outcomes in Iranian healthcare settings. The observed variations in certain subscales highlight the need for healthcare administrators and educators to consider cultural norms and professional perceptions when implementing family engagement strategies. Additionally, recognizing the nuanced ways in which Iranian nurses interpret concepts such as “support” and “burden” can inform training programs and policies aimed at fostering effective nurse–family collaboration.

### 4.1 Limitations of the Study

Several methodological and contextual limitations should be acknowledged. First, the relatively lower reliability observed in two subscales and suboptimal AVE values indicate potential measurement weaknesses. Second, the sample for test–retest reliability was limited, which may affect the stability of the findings over time. Third, the presence of correlated residuals in CFA suggests possible overlap in item content, indicating that some items may not be entirely distinct conceptually. Finally, the study was conducted within specific regions and clinical settings, which may limit the generalizability of the results to broader populations of Iranian nurses.

### 4.2 Recommendations for Future Research

Future studies should aim to address these limitations by expanding the sample to include nurses from diverse regions, specialties, and clinical contexts. Further psychometric evaluation using advanced methods such as item response theory or Rasch modeling could improve the precision and discriminatory power of the scale. Qualitative investigations may also be valuable to explore nurses' perceptions of family involvement in greater depth, helping to refine items and reduce conceptual overlap. Longitudinal studies could assess the stability of the factor structure over time and evaluate the impact of interventions aimed at enhancing family‐centered care. Such research will strengthen the utility of the Persian FINC‐NA and support culturally sensitive practices in nursing care.

## 5 Conclusion

This study establishes the Persian version of the FINC‐NA as a valid and reliable tool for assessing Iranian nurses' attitudes toward family involvement in care, with a culturally adapted four‐factor structure reflecting active family participation, engagement and support, burden, and family strengths and communication. While some subscales showed lower reliability, likely due to cultural interpretations of support and burden, the instrument effectively captures key dimensions of family‐centered care. These findings have practical implications: healthcare administrators can use the FINC‐NA to inform policies and guidelines promoting structured family engagement; nursing educators can incorporate it into training programs to enhance understanding of family roles in care; and researchers can apply the tool to evaluate interventions, explore regional or specialty differences, and refine psychometric properties through advanced modeling. Overall, the Persian FINC‐NA provides a culturally relevant framework to guide practice, education, and policy, supporting collaborative, family‐centered care in Iranian healthcare settings.

## Author Contributions

**Amirmohammad Dahouri:** conceptualization; investigation; funding acquisition; writing – original draft; writing – review and editing; visualization; validation; methodology; software; formal analysis; project administration; resources; supervision; data curation. **Mina Hosseinzadeh:** writing – review and editing; supervision. **Mohammad Hassan Sahebihagh:** writing – review and editing; methodology. **Fereshteh Mostafazadeh Meinag:** data curation.

## Funding

This study was financially supported by Tabriz University of Medical Sciences (Grant No. 74549). The funding body had no role in study design, data collection, analysis, interpretation, or manuscript preparation and submission.

## Ethics Statement

Ethical approval for this study was obtained from the Ethics Committee of Tabriz University of Medical Sciences (Approval ID: IR.TBZMED.REC.1403.332). The study was conducted in accordance with the ethical principles outlined in the Declaration of Helsinki and relevant institutional guidelines. Additional permissions were obtained from the Research Council and the Vice‐Chancellor for Research at the Faculty of Nursing and Midwifery, as well as from hospital administrations. The ethics approval certificate is provided in the supplementary material file 1.

## Consent

Informed consent was obtained from all participants prior to data collection. Participation was voluntary, and all participants were informed about the study objectives, procedures, and their right to withdraw at any time without consequence.

## Conflicts of Interest

The authors declare no conflicts of interest.

## Supporting information


Supporting Information S1

Supporting Information S2

## Data Availability

The data that support the findings of this study are available on request from the corresponding author. The data are not publicly available due to privacy or ethical restrictions.
