# Supplementary material for: Cultural Adaptation and Measurement Properties of the Iranian Version of the Families' Importance in Nursing Care—Nurses Attitudes Questionnaire Based on COSMIN Checklist: A Methodological Study
Source: Int J Methods Psychiatr Res. 2026 Jun 30;35(3):e70093. doi: 10.1002/mpr.70093 (PMC13316964; doi:10.1002/mpr.70093)
Supplement: Supplementary file 1 — Supporting Information S1 [file MPR-35-e70093-s002.pdf]

Tabriz University of Medical Sciences

Research Ethics Committees Certificate

<table>
<tr><td>Approval ID:</td><td>IR.TBZMED.REC.1403.332</td><td rowspan="2">Approval Date:</td><td rowspan="2">2024-07-22</td></tr>
<tr><td>Evaluated by:</td><td>Research Ethics Committees of Tabriz University of Medical Sciences</td></tr>
<tr><td>Status:</td><td colspan="3">Approved</td></tr>
<tr><td>Approval Statement:</td><td colspan="3">The project was found to be in accordance to the ethical principles and the national norms and standards for conducting Medical Research in Iran.
Notice:
1. Although the proposal has been approved by the Biomedical Research Ethics Committee, meeting the professional and legal requirements is the sole responsibility of the PI and other project collaborators.
2. This certificate is reliant on the proposal/documents received by this committee on 2024-07-22. The committee must be notified by the PI as soon as the proposal/documents are modified.</td></tr>
<tr><td>Proposal Title:</td><td colspan="3">: Investigating nurses' attitudes towards the importance of the role of families in nursing care in Tabriz hospitals</td></tr>
<tr><td>Principal Investigator:</td><td colspan="3">Name: Amirmohammad Dahouri
Email: amirmohammaddahouri61@gmail.com</td></tr>
</table>

Dr. Bahman Naghipour
Committee Director
Tabriz University of Medical Sciences

Dr. Parviz Shahabi
Committee Secretary
Tabriz University of Medical Sciences
