# Supplementary material for: Cultural Adaptation and Measurement Properties of the Iranian Version of the Families' Importance in Nursing Care—Nurses Attitudes Questionnaire Based on COSMIN Checklist: A Methodological Study
Source: Int J Methods Psychiatr Res. 2026 Jun 30;35(3):e70093. doi: 10.1002/mpr.70093 (PMC13316964; doi:10.1002/mpr.70093)
Supplement: Supplementary file 2 — Supporting Information S2 [file MPR-35-e70093-s001.docx]

**Scoring Instructions for the Iranian Version of the FINC-NA**

The Iranian version of the *Families’ Importance in Nursing Care – Nurses’ Attitudes* (FINC-NA) questionnaire is constructed as a summated scale, meaning that the total score for each dimension is obtained by summing the responses to all items within that subscale. Each item is rated on a five-point Likert scale ranging from 1 (strongly disagree) to 5 (strongly agree).

Based on the results of the exploratory and confirmatory factor analyses conducted in the present study, a revised four-factor structure was identified for the Iranian version of the FINC-NA. The subscales are:

1. **Family as an Active Care Partner (Fam-ACP)** – 12 items
2. **Family Engagement and Support (Fam-ES)** – 3 items
3. **Family Burden (Fam-B)** – 2 items
4. **Family Strengths and Communication (Fam-SC)** – 6 items

All items are scored in the same positive direction. Higher scores on each subscale indicate more positive attitudes toward the respective construct (e.g., stronger partnership, engagement, or recognition of family strengths).

For cross-study comparability, it is recommended to report the **original summated raw scores** for each subscale. However, to facilitate comparisons between subscales and within-person analyses, a **linear transformation to a 0–100 scale** can also be applied using the following formula:

Transformed score=$\frac{(Raw scale score-Lowest possible score)}{Possible score range}\times100$

This transformation allows all subscale scores to be expressed on a common metric, where higher values reflect more favorable attitudes toward family involvement in nursing care.

The detailed item-to-factor mapping and scoring range for each subscale are provided below.

| Scoring guide for Iranian version of FINC-NA | | | |
| --- | --- | --- | --- |
| **New Factor (Iranian Version)** | **Item included** | **Raw score range (0–100 Transformed range)** | **Conceptual meaning** |
| Factor 1 – Family as an Active Care Partner (Fam-ACP) | 13, 14, 15, 16, 17, 18, 19, 20, 21, 22, 24, 25 | 12-60 (0-100) | The family’s active participation in care provision, decision-making, and partnership with healthcare professionals |
| Factor 2 – Family Engagement and Support (Fam-ES) | 2, 7, 10 | 3-15 (0-100) | Emotional encouragement and perceived support provided by the family |
| Factor 3 – Family Burden (Fam-B) *Reversed scored items* | 23, 26 | 2-10 (0-100) | Emotional and practical strain perceived by families during care |
| Factor 4 – Family Strengths and Communication (Fam-SC) | 3, 4, 5, 6, 11, 12 | 6-30 (0-100) | Family coping abilities, problem-solving, and communicative interaction with nurses |
| (Items 1, 8, 9 were omitted in your EFA due to low communalities.) | | | |
